# Laser-optics-based method to suppress *Mikania micrantha* growth

**DOI:** 10.1038/s41598-022-24451-8

**Published:** 2022-11-18

**Authors:** Yu-Pin Lan

**Affiliations:** College of Photonics, National Yang Ming Chiao Tung University, No.301, Sec. 2, Gaofa 3Rd Rd., Guiren District, Tainan City, 71150 Taiwan

**Keywords:** Optics and photonics, Physics

## Abstract

*Mikania micrantha* is an exotic and aggressive species that can reproduce asexually and sexually through its germinative stem and its featherlike seeds. Present weeding methods cannot effectively or economically control the spread of *Mikania micrantha*. In this article, we propose a method to suppress the growth and spread of *Mikania micrantha* by applying a high-energy laser beam to penetrate its stem. The threshold penetrating optical intensity is 3.1 W/mm^2^. To optimize the damage to the inner tissue of the stem, which includes the vascular bundle and medulla for transporting organic nutrients, water, and inorganic salt, the absorption spectrum of the tissue and laser beam size are analyzed. According to the absorption spectrum of the tissue and growth mechanism of *Mikania micrantha*, a 455 nm blue laser is used as an irradiated light source. A single beam with two different beam sizes or two laser beams with the same beam size is used to optimize the stem damage. By the time the cumulative energy reaches 15 Joules for a single laser beam with dimensions of 0.81 mm × 0.74 mm, the inner tissue will be damaged 97.5%. We perform laser irradiation on the fresh *Mikania micrantha* grown hydroponically, with the result that all samples withered in 30 days. Therefore, using the method before the flowering season of *Mikania micrantha* can effectively inhibit its reproduction.

## Introduction

**Figure 1:**
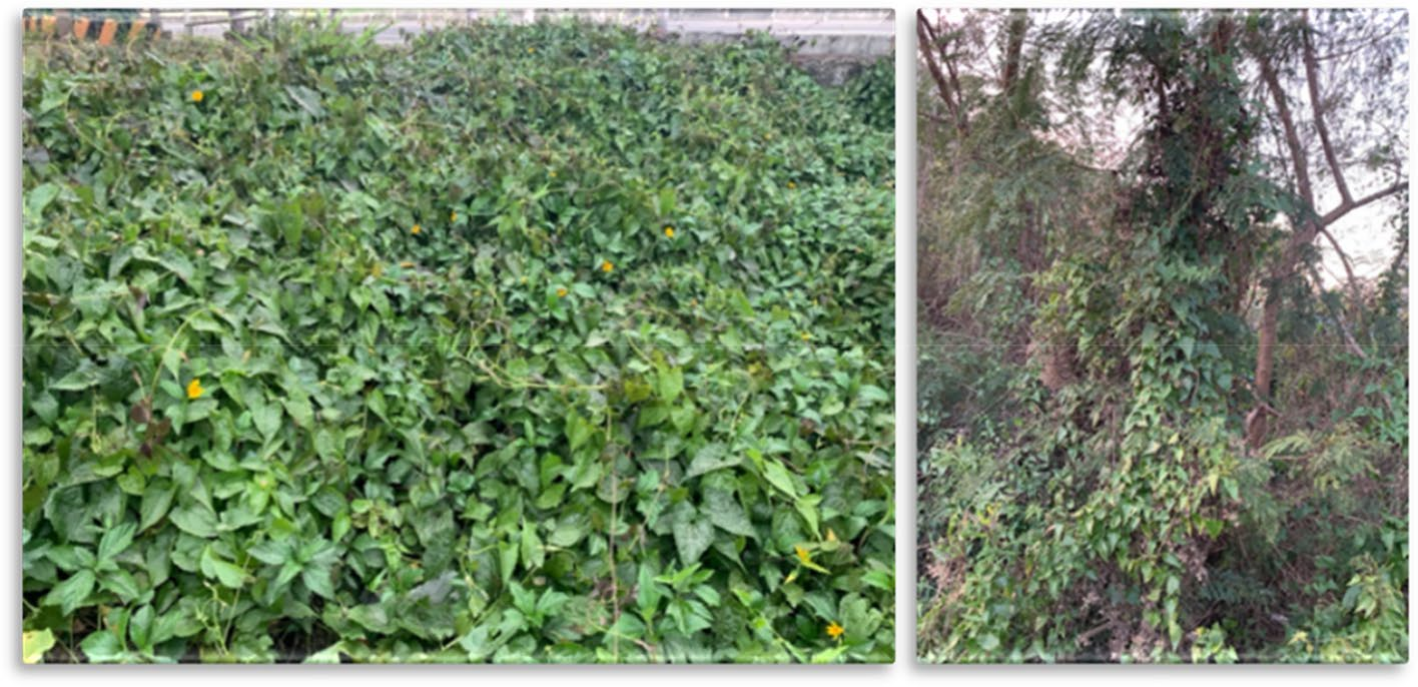
The images show the area and a tree bound heavily populated by *M. micrantha*.

Invasive alien plants harm the diversity and environmental stability of native plants, thereby reducing crop growth and productivity. *Mikania micrantha* (*M. micrantha*) is listed as one of the 100 most invasive species by the International Union for the Conservation of Nature (IUCN)^1^. *M. micrantha* is a perennial twining herb with internodes of 7.5–21.5 cm long. It has opposite leaves and 4 flowers per capitula. A single stalk of *Mikania micrantha* can produce 20,000–40,000 seeds in one season, and can grow vegetatively from its nodes and tiny segments, expanding rapidly^2,3^. Young plants grow quickly at about 8–9 cm per day, and rapidly form a dense cover over entangled leafy stems. Figure 1 shows an area overgrown and a tree covered by *M. micrantha*. Rapid invasion of *M. micrantha* causes damage and economic losses in natural and agricultural environments, leading to a decrease in soil and food stability and alters the nutrient cycle, severely affecting biodiversity and production pipelines^4^.

Several methods to control *M. micrantha* can be proposed. The Consecutive-Cutting Method is applied during at least two consecutive cuts within a 3-week interval before flowering. Three consecutive cuttings result in 91% mortality of the vines. This method reduces the vegetative propagation of *M. micrantha* and has no potential adverse effects on native species. Unfortunately, this control method is very labor intensive and, therefore, uneconomical. Rapid regrowth of cut stumps undermines this method^5–7^. The Flaming Method is a burning technique that helps limit spread and is widely practiced. It does not completely burn the weeds, but effectively suppresses them with heat, destroying the proteins in the plant. Although it is widely used for weeding, it cannot distinguish between weeds and crops. In addition, the weed stock can survive after burning and produce young shoots in a couple of months. A chemical control approach often uses a more effective herbicide because it kills the entire weed. After processing, *M. micrantha* and other vegetation in the treated area are completely wilted^2^. However, chemical herbicides also harm other crops and contaminate the soil. In addition, the other negative impact of using herbicides is greenhouse gas (GHG) emissions^8–10^. Biological control is supposed to be long-lasting, with no adverse effect on nontarget species, and an eco-friendlier solution to control invasive species. Several countries are exploring effective biological control agents for *M. micrantha*^2^. Controversial issues, such as the cost and benefit of the method and the hidden side effects of biological control agents, need to be resolved.

Effective weed control methods to date are based on thermal effects, which increase the temperature to rupture the cell structure, followed by tissue desiccation. Thermal energy can be induced by hot matter or radiation. Heat sources with selectivity and directionality, which are exactly pointed at the location of weeds, can be carried out using lamps, concentrated sunlight, or lasers^11^. Compared to lamps and sunlight, the advantages of lasers for weed control are; high precision direction, weather independence, high customization, and energy variability. Advances in laser technology enable the use of non-diode lasers or diode lasers with a wide wavelength and power range. This opens up new opportunities for photonic weed control. Several groups have controlled the growth in the laboratory using industry laser sources, such as CO_2_ lasers^12–16^, solid-state lasers^15–17^, and diode lasers^15,16,18^. They use lasers to irradiate the apical meristem of mono- or dicotyledonous plants to inhibit their growth^12–18^. *M. micrantha* is a vine without the apical meristem. Since it is easy to grow and distribute by stem nodes, a laser light has to irradiate its other tissue to suppress it. Therefore, we propose a method effectively inhibit *Mikania micrantha* growth and spread by adopting a suitable wavelength and laser energy, and irradiating on a right tissue.

## Materials and methods

### Sample structure

**Figure 2:**
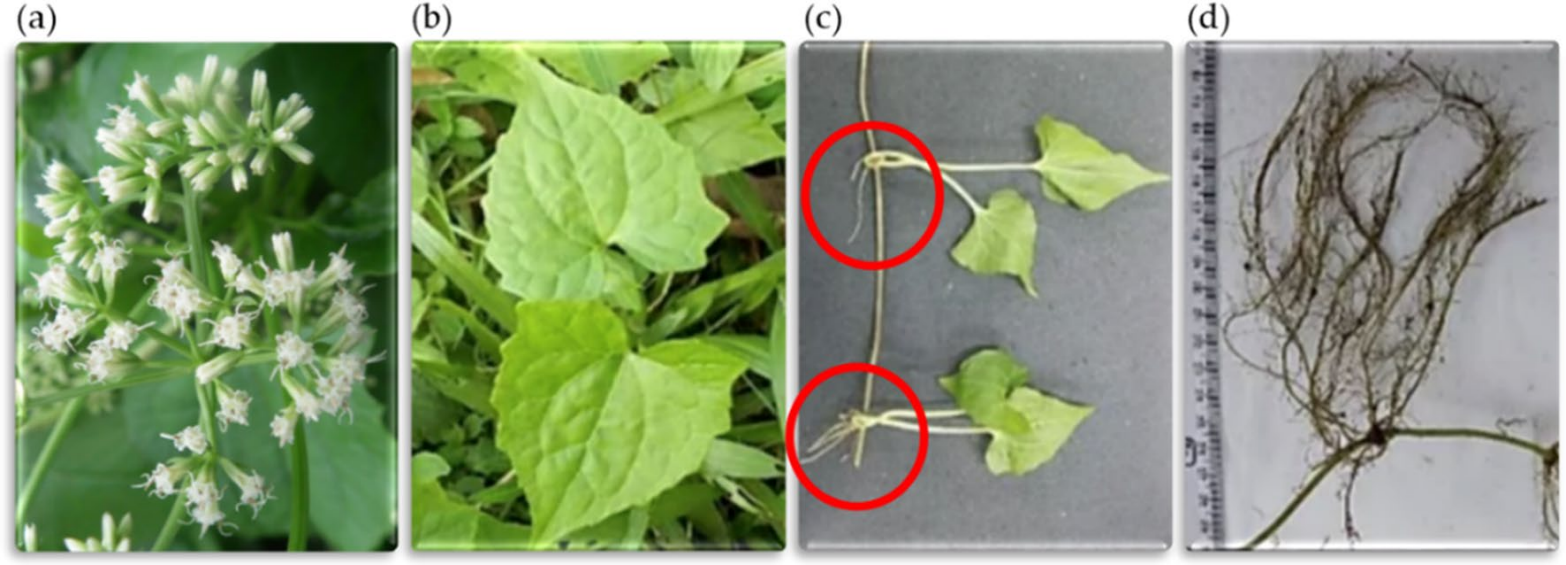
The flowers, leaves, stem, and roots of *M. micrantha* are shown in (**a–d**), respectively.

We collected *M. micrantha* from the land near the College (location is in the south of Taiwan), to grow these samples in a self-build green house. Figure 2 illustrates the flowers, leaves, stems, and roots of *Mikania micrantha*. *Mikania micrantha* is a perennial twining plant with internodes of about 7.5 to 21.5 cm. It has opposite leaves and 4 flowers per capitula, shown in Fig. 2a,b. *Mikania micrantha* can grow vegetatively from the stem nodes, as in Fig. 2c. *Mikania micrantha* is a shallow-rooted plant, shown in Fig. 2d, which quickly entangles with other leafy stem plants and forms a dense cover.

Injury to *M. micrantha* leaves and flowers cannot inhibit its spread due to its ability to reproduce asexually through its germinative stem. As the vegetative fragments (stems, leaves, and ramets) of *M. micrantha* can easily survive, re-sprout, and form new individuals; a practical method to stop *M. micrantha* is weeding before flowering and avoid the stem node from falling to the ground to reproduce asexually. The stem of *M. micrantha* is the key to maintaining growth. The outer to inner structure of the *M. micrantha* stem is divided into the epidermis, cortex, vascular bundle, and medulla. The vascular bundle consists of the phloem and the xylem. The function of the phloem and xylem is to transport organic nutrients, water, and inorganic salt, respectively. If the vascular bundle is damaged, water and nutrients can be interrupted and the germination of new shoots can be prevented. So, we damage the vascular bundle and medulla of the stem to instead of cut it off. We used a laser to increase the temperature of the tissue, then damage the protein to inhibit the growth of *M. micrantha*. To achieve this, we adapted a wavelength and energy density of laser to carefully destroy the phloem and xylem of the plant. This article focuses on optimizing laser energy to the damaged stem area after irradiation. The wavelength selection was based on the absorption spectrum of the stem to perform an effective interaction between light and tissue. To optimize the damaged area, a suitable laser wavelength from the absorption spectrum of the stem, a suitable laser beam size from the optical lens design, and an appropriate illumination method was selected.

### Absorption spectrum

**Figure 3:**
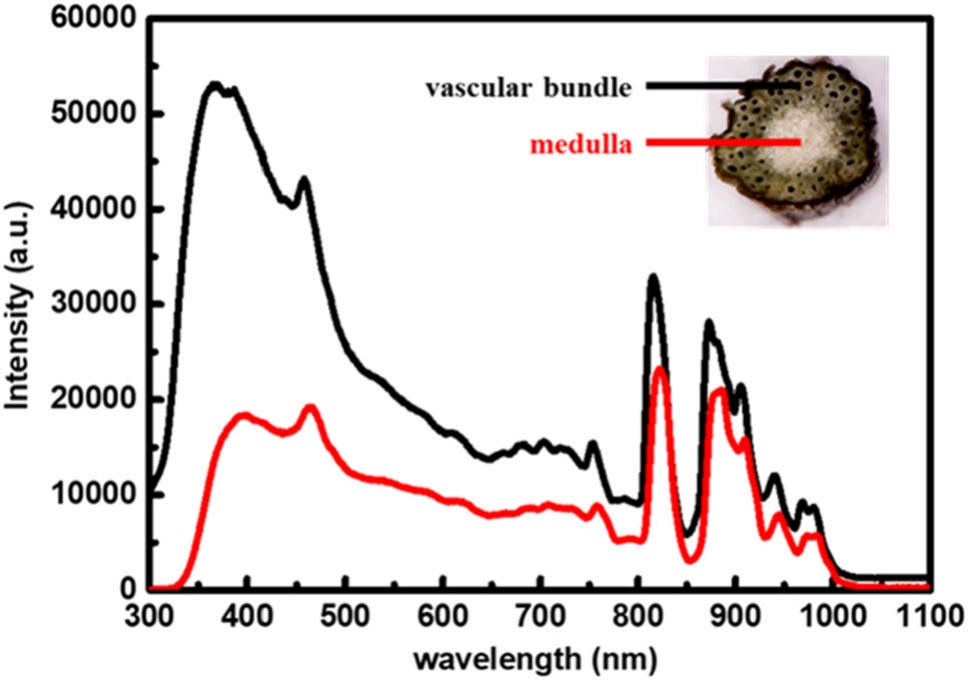
The absorption spectrum of the vascular bundle and medulla in the stem of *M. micrantha*.

To effectively increasing the cell temperature, the laser wavelength must be well chosen, based on the absorption spectrum of the vascular bundle and medulla of the *M. micrantha* stem, as shown in Fig. 3. The absorption spectrum of the vascular bundle and medulla is individually measured by using an optical spectrum analyzer. The vascular bundle shows higher absorption in the blue region, and the medulla is in the near-infrared (NIR) spectrum, but also has a broad absorption band in the blue range. We disregard the NIR region, since wavelengths above 800 nm belong to invisible light. According to the absorption spectrum of the vascular bundle and medulla, the wavelength of 405 nm shows higher absorption. Although 405-nm light has a higher absorption to the stem of *M. micrantha*, it is too close to the UV spectrum. Since most creatures are sensitive to ultraviolet light, we chose a laser with a wavelength of 455 nm as a source to illuminate the stem of *M. micrantha*.

### Experiments

**Figure 4:**
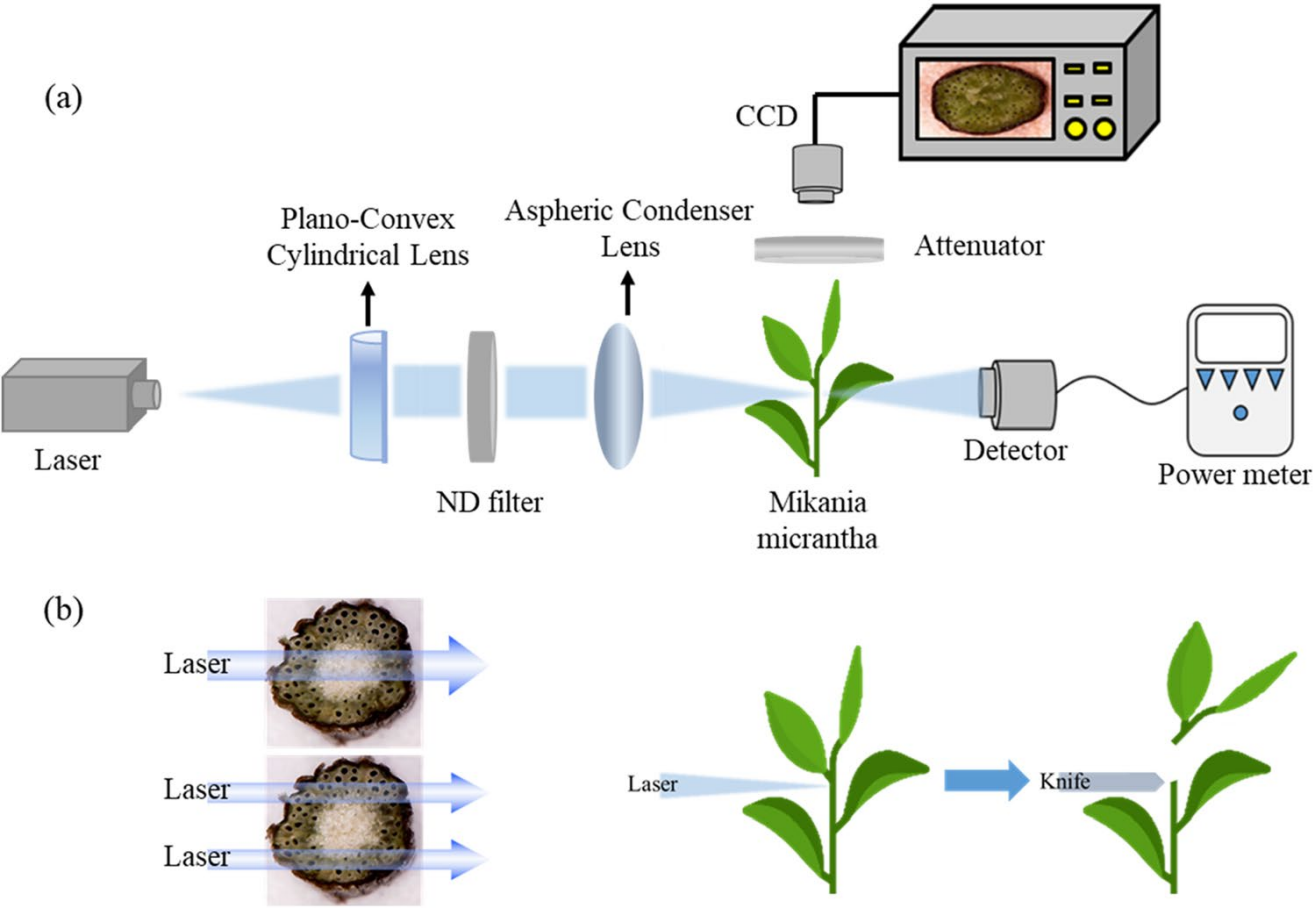
(**a**) Experimental scheme of a laser irradiating *M. micrantha*. (**b**) Schematic diagram of laser irradiated stem and cutting the laser-irradiated stem with a knife.

We conducted the experimental design after considering the convenience and cost of the laser, optimal absorption lines of the stem, and the average stem size of *M. micrantha*. An experimental scheme is shown in Fig. 4a. It consists of a commercial blue-diode laser source with a wavelength of 455 nm. The beam shape of the blue laser diode is elliptical with divergent angles of 9.1 and 1.34 degrees. Lenses were used to change the focus laser beam size on the stem of *M. micrantha* samples. The set of lenses consisted of a cylindrical lens and an aspheric condenser lens. The planoconvex cylindrical lens with a focal length of 12 mm, both sides coated with antireflection of wavelengths from 350 to 700 nm, was used to reduce the large divergent angle to collimate the blue laser beam. The aspheric condenser lens with a focal length of 20 mm and a numerical aperture lens of 0.6 was installed to focus the laser beam on the stem of *M. micrantha*. The laser beam size was measured by the knife-edge method. The minimum beam size is 0.46 mm × 0.42 mm. A continuously variable neutral density filter (ND filter) was placed between two lenses to control the power focused on the weed. A photodetector was used to measure the laser power and the power that passes through the stem. The laser power times the exposure time provided the energy focused on the weed. The photon intensity was calculated in watts per beam area. In addition, a charge-coupled device (CCD) camera was installed to monitor illumination time and the situation of the stem during laser illumination.

The diameters of all fresh *M. micrantha* samples in the experiments are 2 mm. To determine the laser energy requirement for inhibiting the growth of *M. micrantha*, Fig. 4b shows two ways to irradiate the stem of *M. micrantha* in the experiments: one is using a single beam to pass through the medulla of the stem, or pass through the vascular bundle of the stem by using two laser beams. After laser illuminating, we cut the irradiated area of the stem by a knife to observe its cross-section. For further observation of the status of the stem during laser irradiation, we removed the leaves. We irradiated the stem 2 mm below the leaves to observe the impact of laser irradiation on the stem. A CCD was on the top position to observe the variation of the cross-section surface during laser irradiation.

### Ethical approval

Our study complies with relevant institutional, national, and international guidelines and legislation. *Mikania micrantha* was collected from deserted fields on our Campus in this study.

## Results and discussion

**Figure 5:**
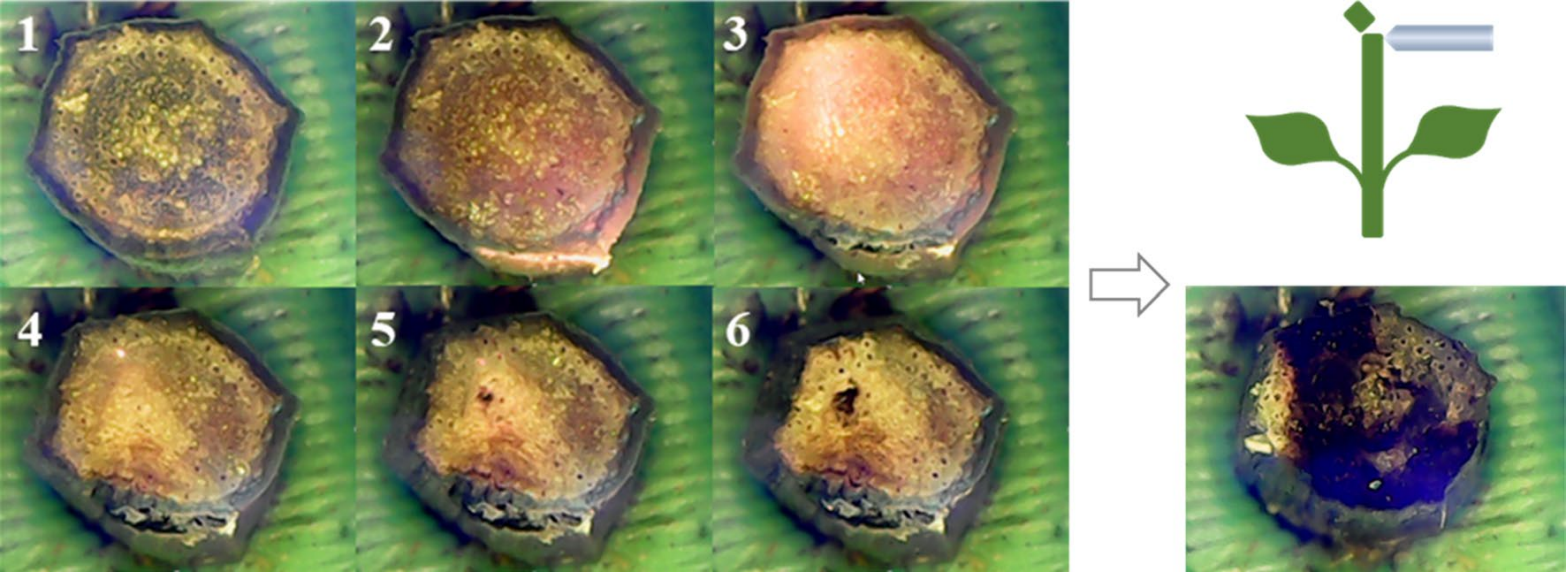
Sequential states of laser illumination on a stem of *M. micrantha*. The cross section of the stem is irradiated by the laser shown in the right bottom.

The CCD records the process of a laser from irradiating to penetrating the stem. Photos 1 through 6 of Fig. 5 show the process of the laser shots on the stem. When the laser started to illuminate a stem, the residual blue light was around the stem. With the exposure time increased, the stem turned red due to the increased temperature of its inner tissue. In photo 3, the stem started to shrink because the photon energy increased the temperature of the localized stem. When the laser penetrated the stem, the illuminated edge of the stem showed carbonization. The high temperature caused the carbonization of proteins. According to photos 4 to 6, the stem was dehydrated, and certain areas looked like blisters caused by burns. The stem shrank during laser exposure. Cut the stem with a knife from where the laser was irradiated. The stem’s inner tissue was burned, almost covering the medulla and vascular bundle. The degree of carbonation on the cut surface indicates that the cells that transport nutrients and water are also damaged. If the stem is fully carbonized, it will break from the stem. Broken stems that fall to the ground may create opportunities for asexual reproduction. In order to prevent the stem from breaking and to block the transport of nutrients and water, it is very critical to control the intensity of the laser-irradiated stem.

### Threshold irradiated intensity

**Figure 6:**
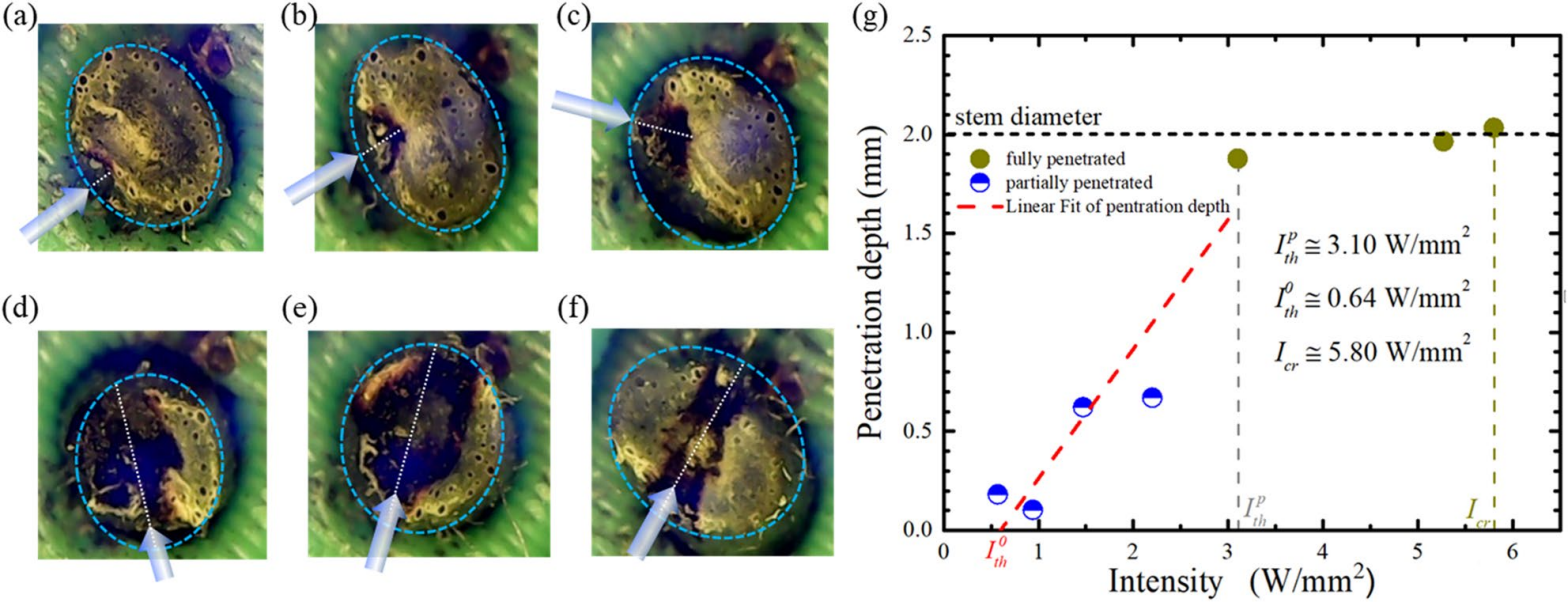
Cross-section status of the stem illuminated at different laser intensity (**a**) 0.57 W/mm^2^, (**b**) 1.47 W/mm^2^, (**c**) 2.2 W/mm^2^, (**d**) 3.1 W/mm^2^, (**e**) 5.27 W/mm^2^, and (**f**) 5.8 W/mm^2^, respectively. (**g**) The penetration depth versus laser intensities.

In addition to the laser power, the laser beam area is also an essential parameter for inhibiting the growth of *M. micrantha*. Laser power divided by the laser beam area is the intensity of the laser. Figure 6a–f show the stem status at different laser intensity illuminations. The blue arrow indicates the direction of laser irradiation. A laser intensity of 0.57 W/mm^2^ damaged only the surface of the stem. When the laser intensity increased to 1.47 W/mm^2^ and 2.2 W/mm^2^, the laser energy penetrated the surface layer and reached the vascular bundle. The white dashed line marked the laser stop location. When the laser intensity increased to 3.1 W/mm^2^, the stems began to be penetrated. It is worth noticing that the laser passes through the stem with both laser intensities of 3.1 W/mm^2^ and 5.27 W/mm^2^. It caused the tissue around the path of the laser to scorch. When the laser intensity rose to 5.8 W/mm^2^, the laser crossed the stem and left an obvious line. Although a higher laser intensity could easily penetrate the stem, the damage region was relatively small. The stem penetration depth could be approximated by a linear relation to intensities, as shown in Fig. 6g. The threshold laser intensity for stem penetration was 3.1 W/mm^2^. For both piercing the stem and damaging the vascular bundle inside, the laser intensity should be between 3.1 W/mm^2^ and 5.27 W/mm^2^.

### Optimization of damaged conditions

**Figure 7:**
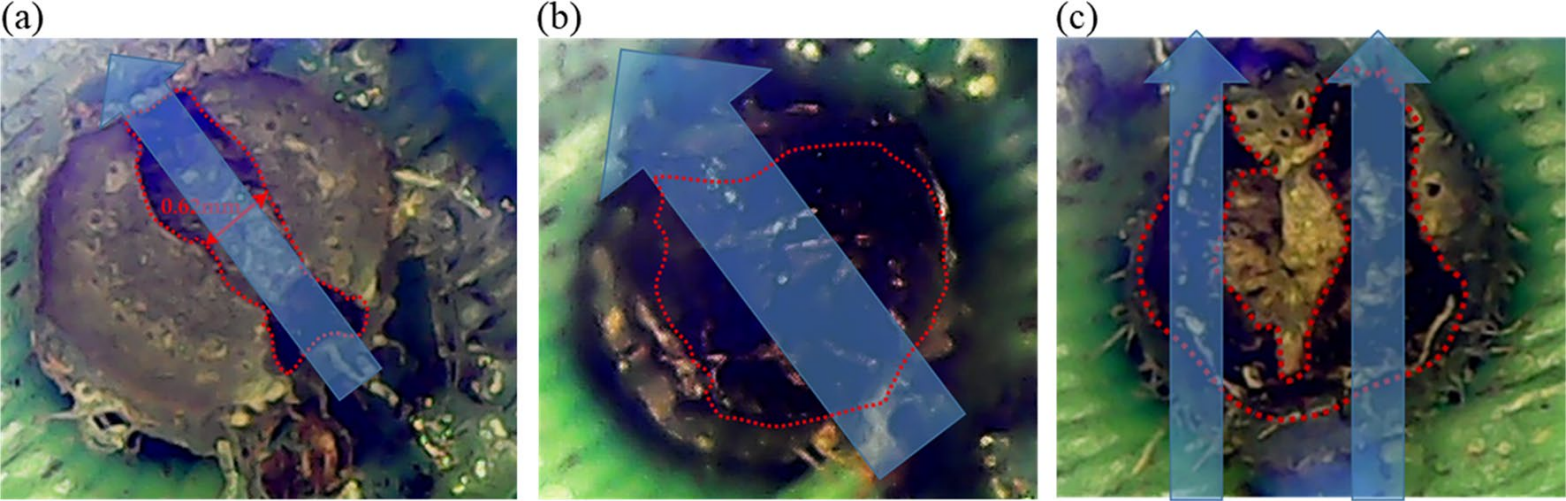
The stem of *Mikania micrantha* was penetrated by a laser marked with a blue arrow, and the damaged area was marked with a red dashed line. (**a**) The laser energy of 2.7 J with a beam area of 0.2 mm^2^, (**b**) the laser energy of 15 J with a beam area of 0.6 mm^2^, and (**c**) two beams with total laser energy of 10.7 J with each beam area of 0.2 mm^2^.

Once the intensity of the laser has been determined, sufficient damage area must be achieved within the stem to allow the growth of the plant to be controlled. Another factor that contributes to efficient plant growth control is the beam size and illumination method of the laser. Use these two parameters to optimize the destructed region of the stems. Figure 7 shows the stem irradiated by a single beam or two laser beams. The blue arrows show laser beams, and the red dashed line marks the damaged area. Figure 7a,b shows the stem irradiated by a single laser beam with two different beam sizes. One laser beam with a beam area of 0.2 mm^2^ was used to irradiate the stem and damage the medulla. When the energy on the *M. micrantha* reached 2.7 J, the laser penetrated the stem, as shown in Fig. 7a, which causes a 0.62 mm wide path through the stem, representing a damaged area of 26.6%. Enlarging the beam area on the stems to 0.6 mm^2^, corresponding to a beam dimension of 0.81 mm × 0.74 mm, accumulated energy of 15 J, resulted in the laser beam passing through the stem. In this condition, the damaged region of the stem grew to 97.5%, which included the medulla and the vascular bundle, as shown in Fig. 7b. Although a small laser beam could penetrate the stem of *M. micrantha* at lower laser energy, the damaged tissue area was too limited to suppress its growth. Figure 7c shows the results of two laser beams that irradiated and penetrated the stem of *M. micrantha*. When the photon energy reached 10.7 Joules, 60.9% of the stem was damaged. Both the medulla and the vascular bundle were burned, which damaged their transport water and nutrients and then inhibits their growth. Although a single laser beam with a large beam size can damage more area of the stem than two laser beams, however, the single laser beam required a longer exposure time to achieve enough energy.

### Performance of fresh plants

**Figure 8:**
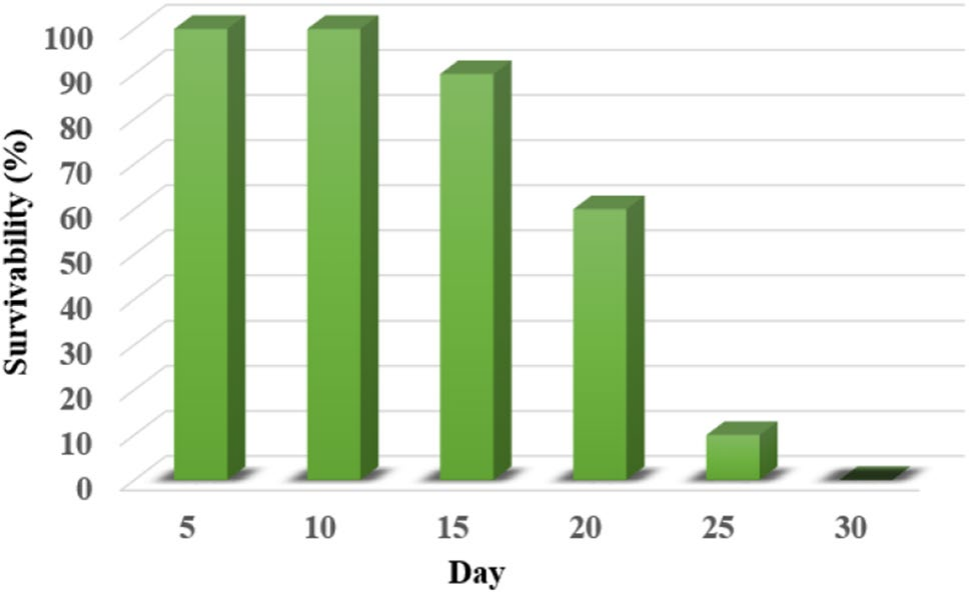
The survivability of *M. micrantha* samples varied with days.

Then, we perform 15 J laser energy with 0.6 mm^2^ beam area to fresh *M. micrantha* samples grown hydroponically in a green house. *M. micrantha* samples with a stem diameter of 2 mm were divided into two groups of 10, the control group without laser irradiation and the experimental group irradiated with laser. The statuses were observed and recorded daily, as shown in the S1—Fig. 1. The first 10 days after laser irradiation, all samples survived. On day 15, one sample died and the leaves of two samples began to turn yellow, and on day 20 four samples withered, as shown in the S1—Fig. 1. All samples withered on the 30th day of the experiment. The survivability of *M. micrantha* samples varied, as shown in Fig. 8. Base on the interaction between a laser and tissue, the temperature of the tissue increases because it absorbs light. A sustained high temperature increases the rate of chemical reactions in plants, reducing or exhausting the concentration of the main metabolic substances. This high temperature causes starvation of the plant, protein decomposition, slow growth, or even stop growth entirely, and some toxic metabolic substances will also be produced. Increased temperatures also result in the loss of water, which causes plants to shrivel^19–21^. If too much water is lost, the cells will dry out and die. On the contrast, all samples of the control group, without laser irradiation, remained live.

## Conclusions

*Mikania micrantha* is a severe invasive species in large parts of Asia. To effectively limit its spread, we use a laser to damage the inner tissue of *Mikania micrantha*, the vascular bundle and the medulla, instead of cutting it off. Based on the absorption spectrum of the inner tissue of the stem and cost, a 455 nm blue laser diode was used as the light source. The penetration depth of the stem depends on laser intensities; the laser intensity for stem penetration has to be greater than 3.1 W/mm^2^. The requirement of damaged energy depends mainly on the efficiency of the energy coupling in the stem of *Mikania micrantha*. High-efficiency energy coupling is achieved through the size of the beam and the duration of irradiation. A single beam with two different beam sizes or two laser beams with the same beam size is used to optimize the stem damage. Using a single beam to irradiate the stem, enlarging the beam dimension to 0.81 mm × 0.74 mm, with accumulated energy of 15 J, achieves 97.5% damaged area of the stem. In contrast, with two laser beams with a beam dimension of 0.46 mm × 0.42 mm irradiating the stem, the photon energy reaching 10.7 Joules, only 60.9% of the stem is affected. Although a large beam size needs more photoenergy to destroy the inner tissue of the stem, it is useful to depress the growth of *M. micrantha*. As the xylem and phloem are functionless after laser illumination, the *M. micrantha* is going to wither due to blockage of water and nutrient transportation. We implemented this method on fresh *M. micrantha* samples and demonstrated that all fresh samples wilt completely within 30 days. In other words, simply irradiating the stems with a laser one month before flowering can stop their growth and spread. With the combination of an intelligent identification system and a laser, automatic work can be performed in the future.

## Supplementary Information

Supplementary Figure 1.

## Data Availability

The data are available from the corresponding author on reasonable request.
