# Supplementary material for: Laser-optics-based method to suppress Mikania micrantha growth
Source: Sci Rep. 2022 Nov 18;12:19864. doi: 10.1038/s41598-022-24451-8 (PMC9674667; doi:10.1038/s41598-022-24451-8)
Supplement: Supplementary file 1 — Supplementary Figure 1. [file 41598_2022_24451_MOESM1_ESM.docx]

Laser-Optics-Based Method to suppress Mikania micrantha Growth

Yu-Pin Lan ^1,^*

Supplementary

Sample A-J presented different samples of Mikania micrantha and their stem irradiated by a laser. S1 -Fig. shows their status during 30 days.


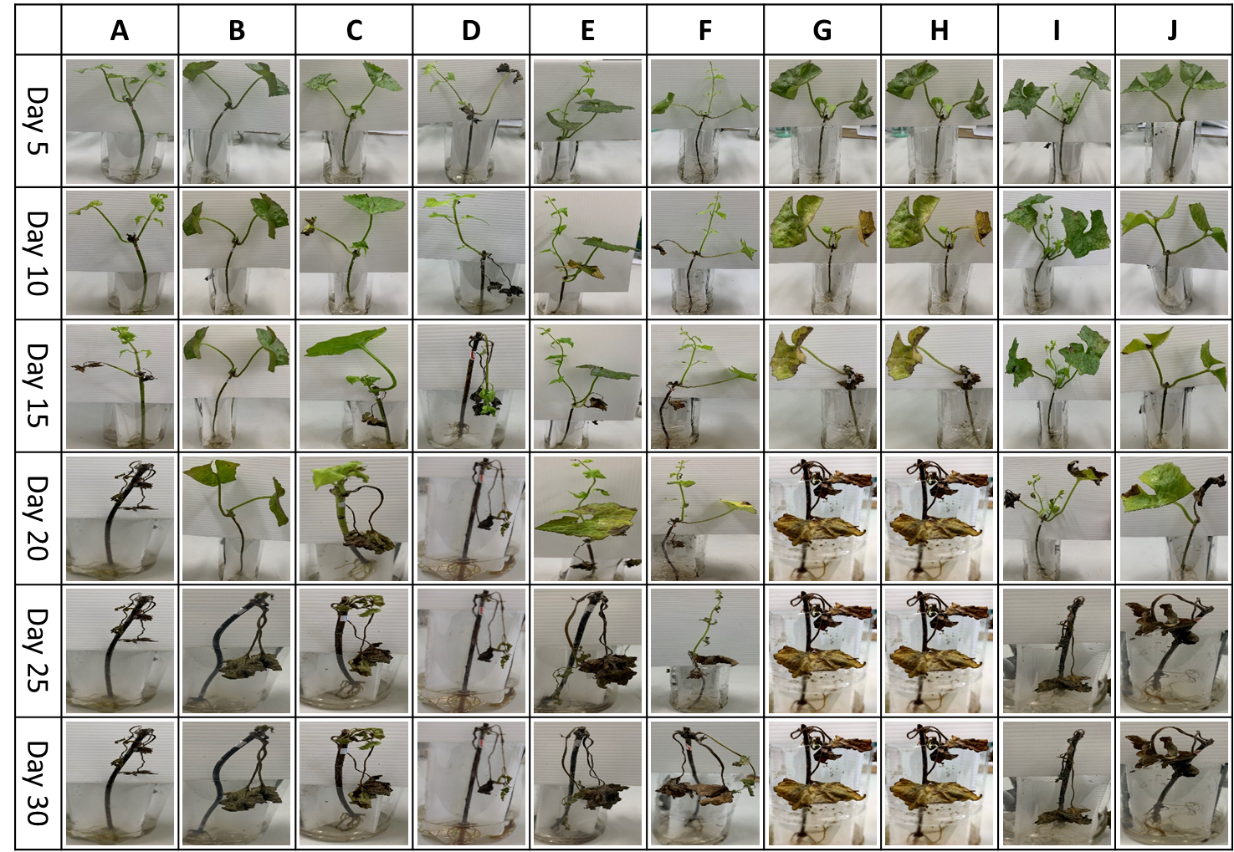


S1 -Fig. 1 A-J Mikania micrantha samples irradiated by a laser were grown hydroponically and the daily status.
